# Selective nourishing of gut microbiota with amino acids: A novel prebiotic approach?

**DOI:** 10.3389/fnut.2022.1066898

**Published:** 2022-12-19

**Authors:** Martin Beaumont, Eugeni Roura, William Lambert, Conny Turni, Joris Michiels, Tristan Chalvon-Demersay

**Affiliations:** ^1^GenPhySE, Université de Toulouse, INRAE, ENVT, Castanet-Tolosan, France; ^2^Centre of Nutrition and Food Sciences, Queensland Alliance for Agriculture and Food Innovation, The University of Queensland, Brisbane, QLD, Australia; ^3^METEX ANIMAL NUTRITION, Paris, France; ^4^Centre for Animal Science, Queensland Alliance for Agriculture and Food Innovation, The University of Queensland, Brisbane, QLD, Australia; ^5^Department of Animal Sciences and Aquatic Ecology, Faculty of Bioscience Engineering, Ghent University, Ghent, Belgium

**Keywords:** prebiotics, amino acids, aminobiotics, gut microbiota, gut health

## Abstract

Prebiotics are dietary substrates which promote host health when utilized by desirable intestinal bacteria. The most commonly used prebiotics are non-digestible oligosaccharides but the prebiotic properties of other types of nutrients such as polyphenols are emerging. Here, we review recent evidence showing that amino acids (AA) could function as a novel class of prebiotics based on: (i) the modulation of gut microbiota composition, (ii) the use by selective intestinal bacteria and the transformation into bioactive metabolites and (iii) the positive impact on host health. The capacity of intestinal bacteria to metabolize individual AA is species or strain specific and this property is an opportunity to favor the growth of beneficial bacteria while constraining the development of pathogens. In addition, the chemical diversity of AA leads to the production of multiple bacterial metabolites with broad biological activities that could mediate their prebiotic properties. In this context, we introduce the concept of “Aminobiotics,” which refers to the functional role of some AA as prebiotics. We also present studies that revealed synergistic effects of the co-administration of AA with probiotic bacteria, indicating that AA can be used to design novel symbiotics. Finally, we discuss the difficulty to bring free AA to the distal gut microbiota and we propose potential solutions such as the use of delivery systems including encapsulation to bypass absorption in the small intestine. Future studies will need to further identify individual AA, dose and mode of administration to optimize prebiotic effects for the benefit of human and animal health.

## Introduction

The gastrointestinal tract is colonized by a complex microbial community composed of hundreds species of bacteria, fungi, protozoa, and yeasts, collectively referred to as the gut microbiota (1–3). In humans like in other monogastric animals, the gut microbiota has major physiological functions for the host, including resistance against colonization by pathogens, degradation of undigested proteins and complex carbohydrates, regulation of nutrient absorption, metabolism, and immunity among others. Disruption of the gut microbiota balance (dysbiosis) has been linked to numerous human diseases such as inflammatory bowel disease, obesity, cancer, diabetes, and autism (4). In farm animals, dysbiosis has also been associated with impairment of the gut development and nutrient absorption, infection by enteric pathogens, inflammation and, ultimately, reduced performance, health, and welfare (2, 4, 5). Diet constitutes the main environmental factor able to modulate the gut microbiota composition and function (6, 7). Dietary constituents may provide competitive advantages to selected microorganisms according to their metabolic requirements and capacities. Complex carbohydrates derived from plants are the main nutrients affecting the gut microbiota and dietary intervention targeting intestinal bacteria have mainly used fermentable fibers, leading to the concept of prebiotics (7).

The term prebiotic was initially defined in 1995 by Gibson and Roberfroid as “a non-digestible food ingredient that beneficially affects the host by selectively stimulating the growth and/or activity of one or a limited number of bacteria already resident in the colon, and thus attempt to improve host health” (8). This definition of prebiotics applied mostly to non-digestible oligosaccharides such as inulin-type fructans and fructo- or galacto-oligosaccarides that promote the growth of *Bifidobacterium* and *Lactobacillus* spp. associated with protection against pathogens and beneficial immunomodulatory and metabolic effects (8, 9). Based on recent advances in the field of gut microbiota, the definition of prebiotics was updated in 2017 by the International Scientific Association for Probiotics and Prebiotics (ISAPP) as “a substrate that is selectively utilized by host microorganisms conferring a health benefit” (10). This expanded definition of prebiotics possibly includes non-carbohydrate substances. For instance, polyphenols are now recognized as a novel class of prebiotics since they are able to provide a health benefit to the host through a modulation of the gut microbiota composition and/or activity (11).

Although less studied than non-digestible carbohydrates and polyphenols, dietary proteins and amino acids (AA) can also influence host health through the regulation of immunity, gut barrier function, oxidative stress but also microbiota composition and its production of bioactive metabolites (12, 13). Thus, this review highlights the potential utilization of AA as a novel class of prebiotics. The structural and chemical diversity of AA represent an opportunity for targeting a broader range of gut bacteria than standard prebiotics by targeting their metabolic requirements/capacities. Moreover, AA are precursors of a broad range of bioactive bacterial metabolites, much more diverse than those derived from saccharides (14). To explore the concept of AA as prebiotics, we first review briefly the metabolism of AA by intestinal bacteria followed by a detailed description of the effects of dietary AA supplementation on intestinal bacteria, metabolites, and the consequences for the host. We also present the potential health benefit of AA supplementation co-administered with beneficial live bacteria (i.e., probiotics). Finally, we discuss technological strategies that may be required to deliver free AA to gut bacteria bypassing the proximal small intestine.

### Amino acid requirements and avoidance in bacterial populations

Host and microbiota-derived proteases hydrolyze dietary and endogenous (host or microorganism-derived) proteins in the intestinal lumen into peptides and AA that can be used by gut bacteria after uptake (14). Available AA can be used by intestinal bacteria for protein synthesis or as carbon and energy sources (15). Luminal AA of dietary and endogenous origin are the main constituents of bacterial protein in the pig ileum, indicating it is likely that *de novo* synthesis of AA by the intestinal bacteria of the foregut of non-ruminants is limited (16). The capacity to synthetize AA differs greatly across bacterial taxa (14). For instance, *Escherichia coli* encodes genes for the biosynthesis of all 20 α-AA while *Lactobacillus* has limited capacity for AA biosynthesis and thus relies on the uptake of extracellular AA (14, 17). It has been proposed that some commensal and pathogenic gut bacteria might have lost biosynthetic pathways for AA due to the high availability of AA in the gut environment (18). It is also important to consider that the autotrophy for AA in gut bacteria can be strain dependent (14). In addition to exogeneous AA, some species of intestinal bacteria such as *E. coli*, use ammonia as the preferred nitrogen source (19). Intestinal bacteria utilize AA in a species-dependent manner, as demonstrated in bacteria derived from the pig microbiota (20, 21). For example, *in vitro* incubation with ^14^C-labeled AA demonstrated that intestinal *E. coli* and *Klebsiella* spp. and *Streptococcus* spp. have differential utilization of AA for protein synthesis (20). The bacterial intracellular AA composition also seems to be species specific compatible with the concept of different AA requirements (22). Interestingly, the saccharolytic pathogen *Campylobacter jejuni* uses a limited range of AA (serine, aspartate, asparagine, glutamate, glutamine, and proline) (23). Further to the roles of protein synthesis and energy source, luminal AA also play an important signaling role in the gut ecosystem, which may have significant impact on the development of enteric pathogens (24). The sensing of arginine by Enterohemorrhagic *E. coli* (EHEC) induces the expression of virulence genes (25). In contrast, the presence of some AA in the medium can impair growth of specific bacteria strains, such as high concentrations of valine and leucine inhibiting *E. coli* growth (26). Overall, the bacterial use of exogenous AA can be classified as nutritionally essential (as a protein building block), non-essential (as energy source), preferred, or avoided/toxic for the growth of specific bacterial species as shown in Table 1.

**TABLE 1:** AA required, utilized, preferred, or avoided according to bacterial species.

Bacteria species	AA required	AA that can be utilized	AA preferred	AA that cannot be utilized	AA avoided or no growth observed	References
**Commensal bacteria**						
*Acidaminococcus fermentans*	Val, Phe, Tyr, Ser, Cys, Arg, His, Trp, Glu	Ala, Arg, Leu, Pro, Thr, Met, Lys, Asp			Pro, Lys, Asp, Asn	(66)
*Bacteroides fragilis*			Asn, Asp, Gln, Glu, Gly, Ser, Thr, His			(67)
*Clostridium sticklandii*		Orn, Lys, His, Asp, Val	Arg, Ser, Thr, Cys, Pro, Gly			(68)
*Lactobacillus arabinosus*	Cys, Met, Trp, Leu. Val, Glu, Thr	Tyr, Phe			Arg	(69)
*Lactobacillus mesenteroides*	Glu, Val					(70)
*Lactobacillus citrovorum*	Glu, Val					(70)
*Lactobacillus mesenteroides*	Glu, Val, Ile					(70)
*Lactobacillus dextranicum*	Glu, Val, Ile					(70)
*Lactobacillus brassicae*	Glu, Val, Ile, Leu, Cys					(70)
*Lactobacillus buchneri*	Glu, Val, Ile, Leu, Cys, Met					(70)
*Lactobacillus pentosus*	Glu, Val, Ile, Leu, Cys					(70)
*Lactobacillus arabinosus*	Glu, Val, Ile, Leu, Trp					(70)
*Lactobacillus brasscitrovorumicae*	Glu, Val, Ile, Leu, Arg, Trp, His					(70)
*Lactobacillus dextranicum*	Glu, Val, Ile, Leu, Cys, His, Thr, Trp, Met					(70)
*Lactobacillus fermenti*	Glu, Val, Ile, Leu, Met, Trp, Phe, Tyr					(70)
*Lactobacillus mannitopoeus*	Glu, Val, Ile, Leu, Met, Arg, Trp, Phe, Tyr					(70)
*Lactobacillus delbrueckii*	Glu, Val, Ile, Leu, Arg, Trp, Cys, Tyr, Ser					(70)
*Lactobacillus casei*	Glu, Val, Ile, Leu, Cys, Arg, Trp, Cys, Tyr, Ser					(70)
*Lactobacillus gayonii*	Glu, Val, Ile, Leu, Arg, Trp, His, Phe, Tyr					(70)
*Streptococcus faecalis*	Glu, Val, Ile, Leu, Arg, Trp, His, Thr, Tyr, Lys, Ala					(70)
*Lactobacillus citrovorum*	Glu, Val, Ile, Leu, Arg, Trp, Cys, His, Thr, Phe, Gly, Ala					(70)
*Lactobacillus pentoaceticus*	Glu, Val, Ile, Leu, Met, Arg, Trp, Cys, His, Thr, Phe, Tyr, Gly, Lys					(70)
*Lactobacillus mesenteroides* + *Lactobacillus brevis*	Glu, Val, Ile, Leu, Met, Arg, Trp, Cys, His, Thr, Phe, Tyr, Gly, Asp, Lys					(70)
*Megasphaera elsdenii*		Ile, Val, Leu	Ser, Thr			(71)
*Streptococcus bovis*		Gln				(72)
*Veillonella* spp.			Arg, Gln, Glu, Lys, Orn			(73)

**Potential pathogens**						

*Campylobacter jejuni*			Asn, Asp, Gln, Glu, Pro, Ser			(74)
*Clostridium difficile*	Gly, Ile, Phe, Thr	Phe, Trp, Arg, Tyr, Ala, Asp, Val	Ser, Met, Leu, Pro		Glu, Lys	(75)
*Clostridium perfringens*			Arg, Ile, Leu, Lys, Thr			(75)
*E. coli*	Ser, Asp, Cys, Asn	Glu, Lys	Asp, Ser		Val, Leu, Ile, Trp, His	(26, 76, 77)
*Fusobacterium nucleatum*			Arg, Asp, Asn, Gln, Glu, Gly, His, Lys, Orn, Thr			(78)
*Fusobacterium varium*				Glu, His, Lys, Ser		(79)
*Klebsiella pneumoniae*			Arg, Asn, Gln, Glu, His, Lys, Met			(20)
*Pasteurella*	Met, Cys, Glu	Leu				(80)
*Peptostreptococcus* spp.			Gln, Leu, Phe, Ser, Thr			(81)
*Pseudomonas*		Ala, Glu, Asp		Met, Cys, Thr		(76)
*Pseudomonas aerunginosa*		Lys				(76)
*Salmonella enterica*		Asp, Glu, Gly, Pro, Ser, Ala, Arg				(82)
*Staphylococcus aureus*	Pro, Arg, Val, Cys, Phe (for enterotoxin production)					(83–85)
*Veillonella* spp.	Lys, Orn					(86)

AA, amino acid; Ala, alanine; Arg, arginine; Asp, Aspartate; Asn, Asparagine; Cys, Cysteine; Glu, glutamate; Gln, glutamine; Gly, Glycine; His, histidine; Ile, isoleucine; Leu, leucine; Lys, lysine; Met, methionine; Orn, ornithine; Phe, phenylalanine; Pro, proline; Ser, Serine; Thr, threonine; Trp, tryptophan; Tyr, tyrosine; Val, valine.

### Metabolite release from amino acid catabolism by intestinal bacteria

The microbial catabolism of AA may impact the host intestinal lumen caused by the release of multiple metabolites resulting from a combination of deamination, decarboxylation, or desulfurization reactions (12). The deamination of AA releases ammonia which may negatively impair mitochondrial respiration, resulting in decreased cell proliferation and barrier function in the intestinal epithelium (27, 28). In addition, the degradation of cysteine by the gut microbiota releases hydrogen sulfide which can reduce mitochondrial respiration and increase inflammation when present at high concentration (29). Microbial catabolism of tyrosine releases *p*-cresol which can induce DNA damages and reduce mitochondrial respiration in intestinal epithelial cells and has been implicated in renal, cardiovascular and neurological disorders (30). Imidazole propionate, a bacterial metabolite derived from histidine, was shown to disrupt insulin signaling and was implicated in diabetes (31). Based on these observations, AA fermentation (also called putrefaction) has often been considered detrimental for health (32).

In contrast, other bacterial metabolites derived from AA were shown to have beneficial effects for host health. Deamination and decarboxylation of glutamate, threonine, alanine, lysine, glycine, and aspartate produces short-chain fatty acids (SCFA; acetate, propionate, and butyrate) which have protective effects against infection and enhance the gut barrier function and immunity (12). The microbial catabolism of branched-chain AA (BCAA) releases branched chain fatty acids (isovalerate, isobutyrate, isocaproate, and 2-methylbutyrate) that can serve as an energy substrate for epithelial cells and promote the barrier function *in vitro* (12, 13, 33). Valerate, a bacterial metabolite derived from proline but also from propionate and ethanol, directly inhibits the growth of *Clostridioides difficile* but not of commensal bacteria (34). Decarboxylation of arginine and lysine produces the polyamines agmatine, spermine, and cadaverine, respectively (35). These metabolites influence mitochondrial function, epithelial proliferation, gut barrier function, and have trophic effects on the developing gut (35). However, the specific effects of gut microbiota-derived polyamines has not been clearly distinguished from those derived from the diet or from host cells and detrimental effects of polyamines have also been described (36). The histidine-derived metabolite histamine reduced the secretion of pro-inflammatory cytokines *ex vivo* (37). The gut microbiota also produces numerous catabolites from tryptophan, including indole, indole-3-propionate, indole-3-aldehyde (38). These bacterial metabolites have protective effects for the gut barrier since they reduce epithelial permeability and inflammation (38, 39) and can also contribute to the effects of the gut microbiota on the brain (40). Interestingly, neurotransmitters (GABA, histamine, serotonin, dopamine) which may have an important role in the gut-brain axis, can also be produced by the gut microbiota through AA catabolism (14). Thus, many AA-derived bacterial metabolites have protective effect on host health.

In summary, AA are used by intestinal bacteria in a species (or strain) specific manner, which highlights the potential of specific AA used as prebiotics for promoting the selective growth of beneficial over pathogenic gut microbes. Moreover, stimulating the production of protective AA-derived metabolites by the gut microbiota has the potential to mediate beneficial effects of AA used as prebiotics on host health.

### Dietary amino acids as modulators of the gut microbiota and consequences for host health

Modification of dietary protein intake would be the most straightforward approach to modify AA supply to the gut microbiota. Increasing protein intake results in a larger amount of dietary protein reaching the distal part of the gut where the microbiota is mostly located (14, 41, 42). The digestibility and AA profiles of dietary protein also influences the availability of AA for the gut microbiota. In general, plant are less digestible than animal proteins and the biological value of the AA composition is commonly higher in the latter compared to the first (43). Modifying dietary protein intake in terms of quality and quantity can also change AA availability for the gut microbiota, which might have both detrimental and beneficial consequences for health (44, 45). It has been suggested in the literature that casein or whey protein, particularly rich in BCAA, could protect against obesity and modulate microbiota in humans and rodents. Similarly, lean seafood or meat with high amounts of aromatic acids, glycine, and taurine could be associated with increased energy expenditure, anti-obesogenic effect, and modulation of microbiota (44). It is also well described in the literature that high-protein intake has been associated with negative effects on gut health, such as inflammatory bowel disease in humans and postweaning diarrhea in piglets (46). In contrast, reducing crude protein level in piglet’s diets has been associated with reduced diarrhea score as reported in a recent meta-analysis (47). As discussed above, pathogenic, and commensal bacteria utilize AA in a specific manner and detrimental or protective metabolites are produced from AA by the gut microbiota. Thus, changing protein intake does not facilitate a targeted supply of AA to the gut microbiota to promote health. Alternatively, dietary supplementation with specific AA is a targeted approach with potential beneficial consequences for host health through a selective growth promotion of beneficial bacteria and through the production of protective metabolites.

Accumulating evidence is showing that dietary supplementation with free AA can modulate the microbiota composition and activity both *in vitro* (33) and *in vivo* (Tables 2–4). This is associated with consequences for the host as reported in mice and humans (Table 2), pigs (Table 3), and chickens (Table 4). Most studies reveal an effect of AA supplementation on the gut microbiota diversity or composition. Thus, the comparison between studies presented in Tables 2–4 does not reveal general trends regarding the effects of AA supplementation on the gut microbiota. The divergence of the results obtained after AA supplementation can be explained by multiple differences between studies including the AA tested, host species, gut segment, dose, and duration of AA supply and exposition or not to an inflammatory or infectious challenge. It is interesting to notice that some effects of the AA supplementations were observed in the large intestine, despite that free-AA are anticipated to be absorbed mostly in the upper part of the intestine. It can be hypothesized that a high level of free-AA might temporally overwhelm the absorptive capacity of the small intestine. A surprising observation is that no linear dose-dependent effects were observed on the microbiota in studies testing the same AA at different level of supplementation (Table 3). Thus, further studies are clearly needed to define more clearly what are the effects of AA supplementation on the gut microbiota. This future work should help determine which AA have the most favorable effects on the gut microbiota and what is the effective level of supplementation. In addition, only few studies presented in Tables 2–4 have investigated the effects of AA supplementation on the production of AA-derived metabolites, the latter being potentially more predictable than the effects on the composition of the microbiota due to functional redundancy (i.e., different bacterial communities can express similar metabolic capabilities).

**TABLE 2:** Effect of free amino acid supplementation on microbiota and health outcomes in humans and mice.

AA	Supplemental level as compared to control	Subject	Duration (d)	Segment	Phylum	Genus	Species	Diversity	Health	References
**Gln**	30 g/d	Overweight or obese patients	14	Feces	↓ *Actinobacteria Firmicutes*	↓ *Pseudobutyrivibrio Veillonella, Dorea, Dialister*	NA	NA	NA	(87)

**BCAA**	14 g twice daily	Hemodialysis patients	120	Feces	−	−	↓ *Bifidobacterium dentium Lacticaseibacillus paracasei*	=	=	(88)

**Arg**	0.5%	Mice	14	Jejunum	↑ *Bacteroidetes* ↓ *Firmicutes*	↑ *Lactobacillus*	NA	NA	↓jej TLR-6, Crs4c, Spla2 ↑jej TLR-8, IFN-γ, MUC2, MUC4, J-Chain, Cryptdin 1, 4, 5, Crsc1c, Ang4	(89)
									↑ il TLR-4, TLR-6, TLR-8, IL1-β, TNF-α, IFN-γ, J-Chain, Cryptdin 1, 4, 5, Crs1c, Ang4, Reg3γ, Lyz2
**Arg**	0.5%	**Mice**	14	Ileum	↑ *Bacteroidetes* ↓ Firmicutes	↑ *Streptococcus* ↓ *Lactobacillus*	NA	NA		(89)

**Asp**	0.5, 1, 2%	Mice	14	Ileum	=	NA	NA	NA	↑ il Cryptdin-1 (2%), PigR (2%)	(90)
									↓ il IL-17 (1%), IFN-γ (1%), Muc2 (0.5, 1%)2
**Asp**	0.5, 1, 2%	Mice	14	Feces	↓ *Firmicutes: Bacteroidetes (0.5%, 1%)* ↑ *Firmicutes: Bacteroidetes (2%)*	NA	NA	NA		(90)

**Gln**	1%	Mice	14	Jejunum	↓ *Firmicutes*	↑ *Streptococcus* *Bifidobacterium*	NA	NA	↑ jej MUC4, Cryptdin 1, 4, 5, Reg3γ ↓jej Crs4c	(91)
									↑ il TLR-4, Reg3γ, Cryptdin-4, IL1-β, TNF-α, IL-17 ↓ il TLR-5
				Ileum	↓ *Firmicutes*	↓ *Streptococcus* *Lactobacillus*	NA	NA		

**Trp**	0.1%	Acetic acid-induced colitis mouse model	7	Colon	=	↓ *Turicibacter* *Candidatus* *Clostridium* *Coprococcus*	NA	=	↑ col TNFα, IL23 ↓ col IL-22	(92)

AA, amino acid; Arg, arginine; Asp, Aspartate; BCAA, branched-chain amino acids; Gln, glutamine; Trp, tryptophan; NA, non-available; jej, jejunum; il, ileum; col, colon; Ang4, angiogenin 4; Crs, cryptdin-related sequence; IL, interleukin; IFN, interferon; Lyz2, Lysozyme 2; PigR, polymeric Ig receptor; Reg3γ, regenerating islet-derived 3g; Spla2, secretory group II A phospholipase A2; TLR, toll-like receptor; TNF, tumor necrosis factor; ↑, significantly increased compared to control group; ↓, significantly decreased compared to control group; = , similar to control group.

**TABLE 3:** Effect of free amino acid supplementation on microbiota, performance and health outcomes in pigs.

AA		Supplemental level (%) as compared to control	Age (d) or BW at start	Duration (d)	Segment	Phylum	Genus	Diversity	Metabolites	Performance and health	References
**Glu or MSG**	MSG	3	25 ± 1.3 kg	30	Jejunum	=	↓ *Prevotella* *Peptostreptococcus* *Clostridium coccoides*	NA	NA	= Perf	(93)
							
	MSG	3	25 ± 1.3 kg	30	Ileum	*↑ Firmicutes* *Bacteroidetes*	↑ Prevotella	NA	NA	=Perf	(93)
							
	MSG	3	25 ± 1.3 kg	30	Cecum	=	↑ *Roseburia*	NA	NA	=Perf	(93)
							
	MSG	3	25 ± 1.3 kg	30	Colon	*↑ Firmicutes*	↑ *Faecaliabacterium prausnitzii Fusobacterium prausnitzii* ↓ *Peptostreptococcus productus Methanobrevibacter smithii*	NA	NA	=Perf	(93)

	Glu	1	77.1 ± 1.3 kg	60	Colon	NA	=	=	↑ *Propionate*, *Valerate*	↓ Body fat	(94)

	Glu	0.5	28 days	28	Ileum	=	*↑ Prevotella* *Anaerovibrio* ↓ *Clostridium* *Terrisporobacter*	=	NA	= Perf ↑ Duo Goblet cells, Villus height/crypt depth ↑ Jej Goblet cells ↑ Il Villus area, Claudin 1, 2, 3, occludin, muc1, IL1β, IL-6, IFNγ, MCP1 ↓ Il TNFα ↓ Ser IL1β	(95)

**BCAA**	BCAA	0.6	28 days	14	Colon	NA	NA	=	=	↑: BWG ADFI ↓: FCR ↑ Duo Villus height ↑ Jej Villus height ↑ Il Villus height	(96)
	
	Leu	1	77.1 kg	60	Colon	↑ Firmicutes, Actinobacteria	↑ *Lactobacillus* *Coriobacteriaceae Collinsella* ↓ *Ruminiclostridium, Clostridiales_vadinBB60*	=	↑ Butyrate Propionate	↓ Body fat, cholesterol, triglycerides ↑ HSL, CPT-1 (adipose tissue)	(97)
	
	BCAA	1.94	28 days	28	Feces	=	=	=	↓5-aminovaleric acid (blood)	↑: Feed intake Energy expenditure	(98)

**Arg**		1	77.1 ± 1.3 kg	60	Colon	NA	↓ *Actinobacteria*	=	↑Valerate	NA	(94)

**Trp**		0.2, 0.4	24	28	Cecum	↑ *Bacteroidetes* ↓ *Firmicutes*	↑ *Prevotella (0.2%)* *Roseburia (0.2%)* *Succinivibrio (0.2%)* ↓ *Clostridium sensu stricto (0.4%)* *Clostridium XI (0.4%)* *Lactobacillus (0.4%)*	↑ (0.2%)	↑ Isobutyrate (0.2, 0.4%) Isovalerate (0.2, 0.4%) ↑ IAA (0.4%)	↑: BWG ADFI = : FCR ↑ Cec Ahr (0.2, 0.4%), CYP1A1 (0.2, 0.4%) ↓ Cec IL-8 (0.4%), TNFα (0.2, 0.4%) ↑ Col Ahr (0.2, 0.4%), CYP1A1 (0.2, 0.4%), CYP1B1 (0.4%), ZO-1 (0.2, 0.4%), Occludin (0.2%) ↓ Col IL-8 (0.4%)	(99)
							
**Trp**		0.2, 0.4	24	28	Colon	NA	NA	NA	↑ Propionate (0.2, 0.4%) Isobutyrate (0.2%) Isovalerate (0.2%) Tryptamine (0.2, 0.4%) ↑ IAA (0.2, 0.4%) ↑ Indole (0.2%)		(99)
	
**Trp**		0.2, 0.4	24	28	Jejunum	↓ *Firmicutes* *Bacteroidetes* ↓ *(0.2%)*, *↑ (0.4%)*	*↑ Lactobacillus* *Clostridium XI* ↓ *Clostridium sensu stricto* *Streptococcus*	↑ (0.2, 0.4%)	NA	↑ Jej ZO-1 (0.2, 0.4%), ZO-3 (0.2, 0.4%), Claudin-1 (0.2, 0.4%), Occludin (0.4%), beta defensin-2 (0.2, 0.4%), beta defensin 3 (0.2, 0.4%), sIgA (0.2%)	(100)
	
**Trp**		0.21 vs. 0.27	24	21	Jejunum	NA	NA	=	NA	NA	(101)
			
**Trp**		0.21 vs. 0.27 Mildly ETEC susceptible	24	21	Jejunum	NA	NA	↑	NA	NA	(101)
	
**Trp**		0.21 vs. 0.27 ETEC-susceptible	24	21	Jejunum	NA	NA	↑	NA	NA	(101)

AA, amino acid; Arg, arginine; BCAA, branched-chain amino acids; Glu, glutamate; Gln, glutamine; Leu, leucine; MSG, monosodium glutamate; Thr, threonine; Trp, tryptophan; Na, non-available; Duo, duodenum; jej, jejunum; il, ileum; col, colon; Ser, serum; Perf, performance; BW, body weight; BWG, body weight gain; ADFI, average daily feed intake; FCR, feed conversion ratio; Ahr, aryl hydrocarbon receptor; ETEC, Enterotoxigenic E. coli; CYP1, Cytochrome P450, family; CPT-1, carnitine palmitoyl transferase-I; HSL, hormone-sensitive lipase; IL, interleukin; IAA, indole-3-acetic acid; MCP-1, monocyte chemoattractant protein-1; PigR, polymeric Ig receptor; Reg3γ, regenerating islet-derived 3g; SigA, secretory immunoglobulin A; Spla2, secretory group II A phospholipase A2; TLR, toll-like receptor; TNF, tumor necrosis factor; ZO, zonula occludens; ↑, significantly increased compared to control group; ↓, significantly decreased compared to control group; = , similar to control group.

**TABLE 4:** Effect of free amino acid supplementation on microbiota, performance and health outcomes in chickens.

AA	Supplemental level (%) as compared to control	Challenge	Age (d)/BW at start	Duration (d)	Segment	Phylum	Genus	Diversity	Performance and health	References
**Trp**	0.2, 0.4	Transportation stress	21	21	Cecum	NA	*↑ Enterococci* (0.2, 0.4%) *Bifidobacteria* (0.4%) ↓ *E. Coli* (0.2, 0.4%) *Clostridia* (0.2, 0.4%) *Enterobacteria* (0.2, 0.4%) *Campylobacteria* (0.2, 0.4%)	NA	↑ADFI (0.2, 0.4%) Ser serotonin (0.2, 0.4%) ↓ Ser corticosterone (0.2, 0.4%) HSP70 (0.2, 0.4%)	(102)

**Trp**	0.1, 0.2		0	42	Cecum		↑ *Anaerobacter* (d21) Sporoacetigenium (d42) ↓ *Streptococcus* (d21)	= (d21) ↑ (d42)	NA	(103)

**Trp**	0.04, 0.08, 0.12		1	42	Ileo-cecal	NA	↑ *Lactobacillus (0.04%)* ↓ *E. Coli (0.04%)*	NA	*↑A*BWG (0.04%) ↓ FCR (0.04%) ↑ Jej Villus height (0.04%), Villus width (0.04%)	(104)
	
**Thr**	0.08, 0.16, 0.24		1	42	Ileo-cecal	NA	=	NA	=Perf	(105)
	
**Thr**	0.1, 0.3		1	21	Cecum	NA	↑ *Lactobacillus (0.3%)* ↓ *Salmonella (0.3%)* *E. coli (0.3%)*	NA	= Perf ↑ Jej Villus height (0.3%), goblet cells (0.1, 0.3%), Villus height/Crypt depth (0.1, 0.3%), IgG (0.1, 0.3%), IgM (0.1%), sIgA (0.1%) ↓ Jej MDA (0.1, 0.3%) ↑ Il Villus height (0.1, 0.3%), goblet cells (0.1, 0.3%), Villus height/Crypt depth (0.1, 0.3%), MUC2 (0.3%), SIgA (0.3%) ↓ Il IL1β (0.3%), IFNγ (0.3%) ↓ Ser MDA (0.3%)	(106)

**Trp**	0.3		1	23	Ileum		*↑ Lactobacillus*	=	↓ jej Crypt depth	(107)
		
**Arg**	0.3	Salmonella typhimurium	1	23	Ileum	↓ *Proteobacteria*	*↑ Candidatus Arthromitus* ↓ *Escherichia–Shigella*	↓	↓ jej Crypt depth ↓ Ser IL1β, IL-8, LITNF ↓ Jej IL-8 ↑ Jej IL-10	(107)
	
**Arg**	0.12, 0.24, 0.36, 0.48		1	30	Ileum	*↑Firmicutes* (0.24, 0.48%) ↓ *Proteobacteria* (0.24, 0.48%)	*↑ Rombutsia*(0.24, 0.48%) ↓ *Candidatus Arthromitus* (0.24%) *Clostridium sensu stricto* (0.24, 0.48%)	NA	↑BWG (0.12, 0.24, 0.36, 0.48%) ADFI (0.48%) ↓ FCR (0.12, 0.24, 0.36, 0.48%) ↑Jej GSH-PX (0.12, 0.24, 0.36, 0.48%), T-AOC (0.36, 0.48%), HMOX1 (0.24, 0.36%), NRF2 (0.36%), IgG (0.36, 0.48%) ↑Il GSH-PX (0.24, 0.36, 0.48%), T-AOC (0.36, 0.48%), HMOX1 (0.36, 0.48%), NRF2 (0.36%), sIgA (0.24, 0.36, 0.48%) ↓ Jej MDA (0.36, 0.48%) ↓ Il IL1β (0.24, 0.36, 0.48%), MyD88 (0.36, 0.48%), TLR4 (0.48%)	(108)
	
**Arg**	0.4	Clostridium perfringens	1	21	Ileum	*↑ Firmicutes* ↓ *Proteobacteria* *Bacteroidetes Plantomycetes* *Verrucomicrobia* *Nitrospirae* *Acidobacteria* *Chloroflexi*	NA	↓	↑ Jej Villus height ↓ *C. Perfringens* lesion scores	(109)

**Arg**	0.61		1	28	Ileum/cecum	NA	↓ *il, cec C. perfringens*	NA	↑plasma D-Xylose ↑Jej sIgA, Claudin-1, IFN-γ ↓ FD4 flux	(110)
			
**Arg**	0.61	Eimeria + Clostridium perfringens	1	28	Ileum/cecum	NA	↓ *il C. perfringens*	NA	↑plasma D-Xylose ↑Jej sIgA, Claudin-1, IL-10, IFN-γ, NOD1 ↑ Il Occludin, IFN-γ, NOD1 ↓ FD4 flux	(101)

AA, amino acid; Arg, arginine; Thr, threonine; Trp, tryptophan; Na, non-available; Duo, duodenum; jej, jejunum; il, ileum; col, colon; cec, cecum; Ser, serum; Perf, performance; BWG, body weight gain; ADFI, average daily feed intake; FCR, feed conversion ratio; FD4, Fluorescein Isothiocyanate-dextran; GSH-PX, glutathione peroxidase; HMOX1, Heme Oxygenase 1; HSP, heat shock protein; Ig, immunoglobulin; IL, interleukin; MCP-1, monocyte chemoattractant protein-1; MDA, malondialdehyde; NRF2, Nuclear factor (erythroid-derived 2)-like 2; MyD88, Myeloid differentiation primary response 88; NOD, Nucleotide Binding Oligomerization Domain; LITNF, lipopolysaccharide-induced tumor necrosis factor-alpha factor; SigA, secretory immunoglobulin A; Spla2, secretory group II A phospholipase A2; T-AOC, total antioxidant capacity; TLR, toll-like receptor; TNF, tumor necrosis factor; ZO, zonula occludens; ↑, significantly increased compared to control group; ↓, significantly decreased compared to control group; = , similar to control group.

The evaluation of host parameters focused on intestinal barrier function, immunity and metabolism revealed modulations associated with the modification of the gut microbiota induced by AA. However, it is not clear yet if these modifications at the gut level are beneficial for the host, which is a requirement to fulfill the definition of prebiotics. In farm animals, the effects of AA supplementation on the microbiota were associated with either no or beneficial effects on growth performance. An important perspective will be to demonstrate that the modulation of the gut microbiota by AA is directly involved in the effect observed at the host level.

### Combination between amino acids and probiotics (symbiotic approach)

The potential synergistic effect of AA supplementation with probiotics has been investigated. Probiotics are “live microorganisms which when administered in adequate amounts confer a health benefit on the host” (46). Combining AA and probiotics corresponds with the concept of “symbiotic” which is “a mixture comprising live microorganisms and substrate(s) selectively utilized by host microorganisms that confers a health benefit on the host” (48). One potential mechanism is that AA could promote the survival of the exogenous microbial species (i.e., the probiotic) in addition of being used as substrates for their growth. For instance, the catabolism of glutamine has been shown to improve the acid tolerance of *Lactobacillus* (49, 50). Therefore, the association of glutamine and the probiotic bacteria *L. plantarum* was expected to increase its survivability and thereby improve its positive effect on gut health. Accordingly, the authors showed that the combination of glutamine (1–4 mM) and *L. plantarum* decreased the translocation of *E. coli* in weanling rabbit ileal loops (51). They observed a synergistic effect indicating that the provision of the two compounds (AA and probiotics) together was more efficient than the anticipated sum of effects resulting from each compound alone. Other studies investigated the effects of the combination of arginine and *Lactobacillus* in rats with acute liver injury. The authors showed that the co-administration of *L. plantarum* and arginine reduced the hepatocellular necrosis and inflammatory cell infiltration, whereas the effect of individual administration of probiotics or arginine alone had lower effects than the two together (52). The potential mechanism of this synergistic effect may involve the metabolization of arginine by *L. plantarum* into polyamines, nitric oxide or its utilization as an energy source. Another study showed that a supplementation with *L. plantarum* and arginine 10 days before an LPS-challenge induced a synergistic reduction of liver damage and inflammation (53). The authors hypothesized that probiotics could direct arginine toward polyamines rather than NO synthesis by decreasing inflammation level and promoting cell proliferation and healing in the liver (53). Altogether, these results suggest that symbiotics composed of AA and probiotics have the potential to promote health to a larger extent than the simple sum of both dietary supplements alone. Multiple combination of probiotics and AA should be tested in future studies, notably by selecting the most promising association based on the capacity of the probiotic strain to metabolize specific AA (Table 1).

## Discussion

Based on the data presented above, it appears AA supplementation can modulate the gut microbiota composition, its metabolic activity and these effects can be associated with benefits for the host. Thus, we consider that dietary AA supplements could fulfill the requirements to be considered as a novel class of prebiotics, the “Aminobiotics.” Additional studies are still required to support this promising concept since important questions remain open.

First, most of the doses of AA supplementation used in the literature are relatively high, raising the question of whether the observed effects are linked to direct effects of AA on the microbiota or to indirect effects mediated by the host (i.e., after absorption), or a combination of both. For example, it has been shown that AA supplementation can increase the expression of intestinal β-defensin (an endogenous small cationic polypeptide that functions as a broad-spectrum antimicrobial) by blocking nuclear factor kappa-B (NF-κB) and MAPK inflammatory signaling pathways and activating the mammalian target of rapamycin signaling pathway (mTOR) (54, 55). Similarly, several AA have been reported to modulate the secretion of immunoglobulin A (IgA) in the intestine (56, 57) which could in turn affect the gut microbiota. Therefore, it is very likely that dietary AA affect gut microbial composition both directly (i.e., as a substrate) and indirectly (i.e., through modulation of host factors). Second, only nine AA out of the 20 proteinogenic ones have been tested and they were tested only a limited number of times which urges to be cautious when drawing conclusions. Third, microbiota analysis was not always the main endpoint of the studies which can also generate some biases.

Another challenge will be to develop strategies to supply AA reaching the lower gut were most of the microbiota develops. Indeed, when provided in a free form, AA are rapidly absorbed in the proximal small intestine (58). In contrast, the microbiota density is higher in the distal part of the digestive tract, mostly in the ileum, caecum, and colon (59). One potential strategy to circumvent this lack of space/time synchrony between dietary free AA release and microbiota, would be to delay the release of specific free AA by using protective delivery systems such as fat matrix encapsulation. The encapsulation of AA with probiotics may hypothetically optimize their use by the target communities of bacteria. Another possibility to deliver AA to the gut microbiota would be to combine AA with polyphenols that escape absorption in the small intestine. AA bound to polyphenols might potentially reach the colon and be degraded by the gut microbiota. A recent study showed that the supplementation of piglets with a mix of 0.1% AA (L-arginine, L-leucine, L-valine, L-isoleucine, L-cystine) and 100 ppm grape polyphenols increased the concentration in the caecum of bacterial metabolites derived from AA (e.g., isovalerate and 2-methylbutyrate) (49). Moreover, it is important to consider that the intestinal AA absorption capacity by the host is region dependent. Concurrent with higher expression of brush-border exopeptidases in the distal part of the small intestine (60), the absorption capacity for free AA by enterocytes is the highest in mid- to lower small intestine (61, 62). The latter is mediated by a complex system of brush-border Na^+^ dependent and independent transporters with considerable overlap and competition between AA (63). In addition, differences in rate of absorption between AA are noticeable (61). Therefore, any strategy to supply free AA to location-specific microbiota should be carefully designed.

AA supplementation can also be associated with deleterious effects on the gut microbiota. For example, in poultry, it has been reported that the consumption of diets high in glycine such as fish meal or gelatin are associated with increased populations of pathogenic *C. perfringens* (64). In line with these results, a study by Dahiya et al. reported that birds fed glycine at high levels (34.3 or 47.7 g/kg) in an encapsulated form to slowly release the AA along the entire length of the gut exhibited a higher number of *C. perfringens* and a lower number of *Lactobacillus* in the ileum compared to birds fed low levels of encapsulated glycine (7.6 or 21.0 g/kg). This higher colonization was associated with higher intestinal lesions and reduced performance (65). This reinforces the importance of carefully selecting both the AA supplement type and dose to modulate microbiota to deliver a healthy outcome.

## Conclusion

Dietary supplementations with free AA modulate the microbiota composition and metabolic activity in association with consequences for host health. These properties indicate that AA have the potential to be used as a novel class of prebiotics (“Aminobiotics”). The successful utilization of AA as prebiotics to selectively nourish gut microbiota still requires to (i) select the most appropriate AA and dose of supplementation, (ii) develop strategies to deliver the desired AA profile to the microbiota, and (iii) demonstrate that the modulation of the microbiota by AA is directly involved in benefits for host health. Overall, utilization of AA as prebiotics, alone or in combination with probiotics, will expand the nutritional tools available to target the gut microbiota for human and animal health.

## Data availability statement

The original contributions presented in this study are included in the article/supplementary material, further inquiries can be directed to the corresponding author.

## Author contributions

TC-D, WL, ER, JM, and MB contributed to the conception and structure of the manuscript. TC-D, MB, and CT created and organized the tables. TC-D wrote the first draft of the manuscript. TC-D, WL, ER, JM, CT, and MB contributed to manuscript writing and revision. All authors approved the final version for submission.
